# Exploring the Relationship between Spontaneous Sister Chromatid Exchange and Genome Instability in Two Cryptic Species of Non-Human Primates

**DOI:** 10.3390/ani13030510

**Published:** 2023-02-01

**Authors:** Mariela Nieves, Fiona Puntieri, Susan M. Bailey, Marta D. Mudry, David G. Maranon

**Affiliations:** 1 Centro de Investigación en Reproducción Humana y Experimental (CIRHE), Centro de Educación Médica e Investigaciones Clínicas (CEMIC), CONICET, Buenos Aires C1431-CABA, Argentina; 2 Grupo de Investigación en Biología Evolutiva (GIBE), Departamento de Ecología, Genética y Evolución (DEGE), Facultad de Ciencias Exactas y Naturales, Universidad de Buenos Aires, Buenos Aires C1428-CABA, Argentina; 3 Department of Environmental and Radiological Health Sciences, Colorado State University, Fort Collins, CO 80521-1618, USA; 4 Instituto de Ecología, Genética y Evolución de Buenos Aires (IEGEBA), CONICET-Universidad de Buenos Aires, Buenos Aires C1428-CABA, Argentina

**Keywords:** *Ateles*, primates, genome instability, G-SCE, T-SCE, CO-FISH

## Abstract

**Simple Summary:**

Studies on the evolution of Neotropical primates are currently based on molecular cytogenetics. Recent technological advancements have provided new capabilities and opportunities to explore novel mechanisms of evolutionary dynamics. We analyzed the genomic instability and variability in two species of *Ateles* by characterizing the spontaneous frequencies of sister chromatid exchange (SCE) along chromosomes (genomic) and specifically within telomeres. Our analyses support the hypothesis that regions of *Ateles* chromosomes susceptible to recombination events represent fragile sites and evolutionary hot spots. Therefore, we propose SCE analyses as a valuable indicator of genome instability in non-human primates.

**Abstract:**

There are extensive studies on chromosome morphology and karyotype diversity in primates, yet we still lack insight into genomic instability as a key factor underlying the enormous interspecies chromosomal variability and its potential contribution to evolutionary dynamics. In this sense, the assessment of spontaneous sister chromatid exchange (SCE) frequencies represents a powerful tool for evaluating genome stability. Here, we employed G-banding, fluorescence plus Giemsa (FPG), and chromosome orientation fluorescence in situ hybridization (CO-FISH) methodologies to characterize both chromosome-specific frequencies of spontaneously occurring SCE throughout the genome (G-SCE) and telomere-specific SCE (T-SCE). We analyzed primary fibroblast cultures from two male species of *Ateles* living in captivity: *Ateles paniscus* (APA) and *Ateles chamek* (ACH). High frequencies of G-SCEs were observed in both species. Interestingly, G-SCEs clustered on evolutionary relevant chromosome pairs: ACH chromosomes 1, 2, 3, 4, and 7, and APA chromosomes 1, 2, 3, 4/12, 7, and 10. Furthermore, a statistically significant difference between the observed and expected G-SCE frequencies, not correlated with chromosome size, was also detected. CO-FISH analyses revealed the presence of telomere-specific recombination events in both species, which included T-SCE, as well as interstitial telomere signals and telomere duplications, with APA chromosomes displaying higher frequencies, compared to ACH. Our analyses support the hypothesis that regions of *Ateles* chromosomes susceptible to recombination events are fragile sites and evolutionary hot spots. Thus, we propose SCE analyses as a valuable indicator of genome instability in non-human primates.

## 1. Introduction

In the context of nuclear architecture studies, the genome is considered as a chromatin structure whose regulation depends on several superimposed levels of organization, which change over the course of the cell cycle to favor DNA accessibility to replication and repair machinery [1]. Therefore, studying replication in the context of DNA repair allows a better understanding of genome instability and its contribution to large-scale chromosomal rearrangements. In addition, some of these rearrangements occur at fragile sites that are particularly susceptible to breakage. These regions, considered genomic instability “hot spots”, have served to inform variation in chromosome structure and their potential implications for speciation processes, particularly in primates. Therefore, several authors have proposed that biomarkers, such as fragile sites (FS) and interstitial telomeric sequences (ITS), are associated with different degrees of genomic instability and variability generation to ease and/or promote chromosomal evolution [2,3,4]. Over time, technological advances have enabled improved identification of chromosomal rearrangements and assessment of genomic instability. Cytogenetic methodologies in particular have become valuable tools for genome characterization at the chromosomal and molecular levels. Molecular cytogenetics has revealed both simple and complex rearrangements between and within chromosomes and assisted in delineating genomes’ reorganization. For example, Zoo-FISH can accurately detect homologies and rearrangements between the human species and other mammals, such as non-human primates [5,6,7,8,9,10].

Similarly, the genome reorganization that results from the repair of spontaneous and/or induced DNA damage can be analyzed by evaluating frequencies of genomic sister chromatid exchange (G-SCE) using the fluorescence plus Giemsa (FPG) staining technique [11]. Telomeres, the highly conserved and repetitive G-rich “end-caps” of linear chromosomes, are especially vulnerable to recombination/exchange events, which can now be detected using strand-specific chromosome orientation-fluorescence in situ hybridization (CO-FISH) [12,13], allowing for the determination of telomere-specific sister chromatid exchange (T-SCE) frequencies as an indication of instability. Platyrrhini chromosomes (New World monkeys) have been extensively studied, and different patterns of rearrangements involved in chromosomal dynamics and evolution have been proposed [10,14,15,16,17]. However, only a few studies have been published on the potential underlying role of genomic instability [18,19,20,21]. Recently, Puntieri et al. [22] performed an analysis on the genomic instability in four species of Platyrrhini (*Alouatta caraya, Ateles chamek, Ateles paniscus*, and *Sapajus cay*). Spider monkeys (*Ateles*, Atelidae family) are particularly attractive for analyses of chromosomal dynamics and genome instability because, in addition to having one of the lowest diploid numbers of any Platyrrhini (2N = 32–34), they also possess a karyotype with a relatively high proportion of heterochromatin (3–6% of the chromatin) and well-established chromosomal rearrangements [23,24,25,26]. The species chosen for this work, *Ateles chamek* and *A. paniscus* are not any pair of *Ateles* species, they are the two cryptic species of the genus [25]. Therefore, it is even more interesting to compare their genomes and be able to explain the differences. *Ateles* species showed a highly significant proportion of unstable bands mainly found in the rearranged regions, which is consistent with the numerous genomic reorganizations that likely occurred during chromosomal evolution of this genus [17,23,25].

In the present study, we determined the spontaneous frequencies of G-SCEs and T-SCEs in two *Ateles* species living in captivity, *A. chamek* (ACH) and *A. paniscus* (APA), as a means of evaluating genomic instability. Our chromosome-specific analyses of these biomarkers facilitated the characterization of each species’ distribution of SCE along individual chromosomes and within telomeres, and comparison of G-SCE and T-SCE frequencies between the two species of non-human primates. Lastly, and relevant to wildlife management and ex situ conservation, we also begin to establish the baseline of the background genomic instability in both species, which will be useful for future studies on the exposure to xenobiotic agents or environmental stress. 

## 2. Materials and Methods

### 2.1. Biological Material

We used frozen primary non-human fibroblasts isolated from four male individuals. The skin biopsy samples were from three (3) *Ateles chamek* (ACH) and one (1) *Ateles paniscus* (APA) (see Specimen information sheet in Supplementary Material). Following standard defrosting protocol, cell monolayers were maintained at 37 °C until reaching ~85% confluency. Cells were then trypsinized and 1 mL cell suspension re-plated in 7 mL culture medium containing other essential components, including 5 ul/mL bromodeoxiuridine (BrdU; Sigma-Aldrich, Massachusetts, USA) for G-SCE/FPG staining, or 10 uM bromodeoxyuridine/bromodeoxicytodine (BrdU/BrdC; Sigma-Aldrich) for T-SCE/CO-FISH staining. Species status was confirmed following the standard protocol according to Steinberg et al. [27] by analyzing and comparing G- and C-banded metaphases with previously published karyotypes of ACH and APA.

### 2.2. Experimental Development

#### 2.2.1. Characterization of the Cell Proliferation

The number of metaphases in the first (M1), second (M2), and third cell cycle (M3) was determined following FPG staining, and the replication index (RI) was calculated according to the following formula: I = (1 * (M1) + 2 * (M2) + 3 * (M3))/Total number of metaphases [28]. The RI varies between 1 and 3, presenting values close to 1, 2, or 3 when most metaphases analyzed are in M1, M2, or M3, respectively. Likewise, SCE was counted exclusively in M2 cells, i.e., those containing only chromosomes with one dark and light chromatid after FPG staining. SCE frequencies per chromosome and per metaphase were calculated.

#### 2.2.2. Sequential G-Banding and Fluorescence plus Giemsa (G-FPG) Technique to Detect G-SCEs

The protocol repeated the scheme used in previous work [22]. G-banding with a modified Wright staining method was applied to all metaphases as follows: first, slides were maintained for one week at room temperature, in the dark. Then, they were pretreated in 2xSSC for 2.5 min at 65 C, washed with distilled water and covered with a 3:1 Wright stain [Sørensen buffer (KH2PO4 and Na2HPO4 solutions at a 2:1 ratio)] in the dark for 2.5 min. Following the recording of all the available metaphases, slides were destained and treated by the fluorescence plus Giemsa (FPG) technique to visualize SCEs. Briefly, the slides were incubated with Hoechst 33258/2xSSC/distilled water for 20 min, irradiated with 365 nm UV light (Stratalinker 2400) for 35 min and rinsed in deionized water. Then, they were incubated in 2xSSC for 2 h at 60 C, rinsed in distilled water, and counterstained with 4′,6-diamidino-2-phenylindole (DAPI). We scored G-SCEs on cells that had progressed through two rounds of replication (M2) in the presence of BrdU.

#### 2.2.3. Chromosome Orientation-Fluorescence in Situ Hybridization (CO-FISH)

CO-FISH is a strand-specific methodology that can be used to detect rearrangements within specific target sequences, such as telomeres; complementary single-stranded peptide nucleic acid (PNA) probes are hybridized to telomeric sequences selectively rendered single-stranded [12,13]. Briefly, cells were cultured in BrdU/BrdC-containing media for one cell cycle (M1). Then, after 24 h, cells were fixed, the metaphases dropped onto slides, which were stained with Hoechst 33258 for 20 min. Next, the slides containing BrdU/BrdC-substituted DNA were exposed to 365 nm ultraviolet light for 35 min and rinsed in deionized water. Then, nicked DNA was removed via digestion with Exonuclease III for 20 min at 25 °C. Finally, the remaining single-stranded DNA (template/parental strands) were hybridized with a G-rich Cy3-labeled PNA probe (TelG-Cy3- OO-TTAGGGTTAGGGTTAGGG; Bio-Synthesis, Lewisville, Texas) to detect the leading-strand telomeres, which were sequentially followed by the hybridization of a C-rich A488-labeled PNA probe (TelC-Alexa488-OO-CCCTAACCCTAACCCTAA; Bio-Synthesis) to detect lagging-strand telomeres. Lastly, slides were counterstained with 4′, 6-diamidino-2-phenylindole (DAPI) for imaging and analysis [12, with modifications].

### 2.3. Image Acquisition, Processing, and Frequency Analysis

Metaphase images were acquired using a Zeiss Axio-Imager.Z2 microscope that captured fluorescent images with a Coolsnap ES2 camera running Metamorph software (Molecular Devices, Sunnyvale, CA, USA). MetaSystems (GmbH, Altlussheim, Germany) was used for image processing. For G-SCE quantification, a minimum of 20 FPG images per individual were captured and analyzed, and the number of SCEs per chromosome and per metaphase was determined.

**Figure 1:**
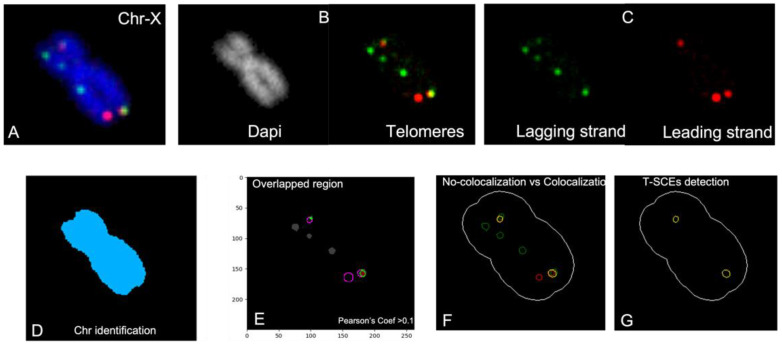
T-SCE analysis: (**A**) Image showing a chromosome counterstained with DAPI (blue), and the leading (red) and lagging (green) strand telomeres. For the detection of T-SCEs we customized a CellProfiler pipeline that identifies chromosomes (**B**) (in grayscale) and telomeres (in red and green). The separation of the channels allowed the software to count **(C**) green signals (lagging strand telomeres) and red signals (leading-strand telomeres). Digitalization of (**D**) chromosomes and telomere signals were required to calculate (**E**) overlapped regions with a Pearson’s coefficient > 10%. The outcome of the analysis shows (**F**) the number of green and red signals, as well as the number of (**G**) colocalized signals representing T-SCEs.

In addition to the absolute number of SCE/chromosome and SCE/cell, and because the two species had different numbers of chromosomes, the number of SCEs relative to the total chromosomes of all metaphases was quantified. The distribution of the SCE frequencies/chromosome was then plotted for each species to characterize the spontaneous induction of G-SCEs. For each species, the mean frequency of the expected SCE was compared to the observed frequency for each chromosome using a chi-square test (statistical package STATISTICA 10). The expected frequencies calculated from the relative size of each chromosome and *p*-values were considered statistically significant at the *p* < 0.05 level. For T-SCE quantification, ImageJ (https://imagej.nih.gov/ij/ (accessed on 15 February 2013)) and CellProfiler (https://cellprofiler.org/ (accessed on 15 February 2013)) were used to analyze the CO-FISH images (in both, the latest required updates were made in 2022). We customized CellProfiler’s pipeline to recognize individual chromosomes in each metaphase and classify and count each telomere (red or green). CellProfiler identified T-SCE as colocalized signals using Pearson’s coefficient with an overlap > 10 percent (Figure 1). Finally, we compared T-SCE and G-SCE frequencies and the mean sample frequencies between groups using one-way ANOVA with Dunn’s post hoc analysis (Prism 9 GraphPad).

## 3. Results

### 3.1. Characterization of the Cell Proliferation

**Figure 2:**
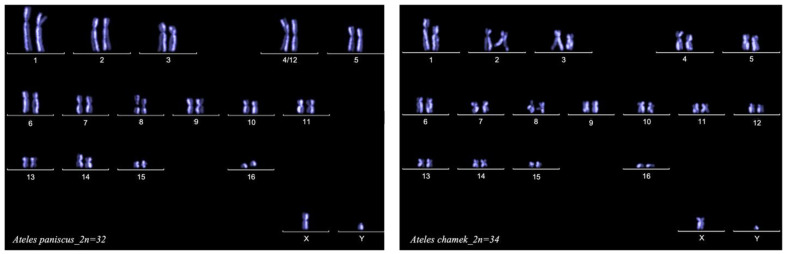
Harlequin staining pattern of chromosomes arranged in the typical karyotypes for *A. paniscus* and *A. chamek*.

To calculate the replication index (RI) for each species (*Ateles chamek* and *Ateles paniscus*), we quantified the numbers of first (M1), second (M2), and third (M3) cycle metaphases after 72 h in culture. Both species showed values close to 2, indicating that most cells went through two cycles of cell division during the culture time. Figure 2 illustrates the typical “harlequin” staining pattern after two cell cycles of replication in the presence of BrdU and FPG staining; chromosomes are arranged in the karyotype of each species.

### 3.2. Analysis of the Genomic Sister Chromatid Exchange (G-SCE)

From the analysis of the M2 metaphases following FPG staining, frequencies of the absolute number of G-SCEs per cell and number of G-SCEs relative to the total number of chromosomes from all metaphases scored were calculated (Table 1). Both ACH and APA showed relatively high frequencies of spontaneous G-SCEs, as compared to reported values for other primates [19,20,21,22] (see Suppl Table S1 sheet in Supplementary Material). Differences between the two species in both absolute and relative G-SCE frequencies were also observed.

**Figure 3:**
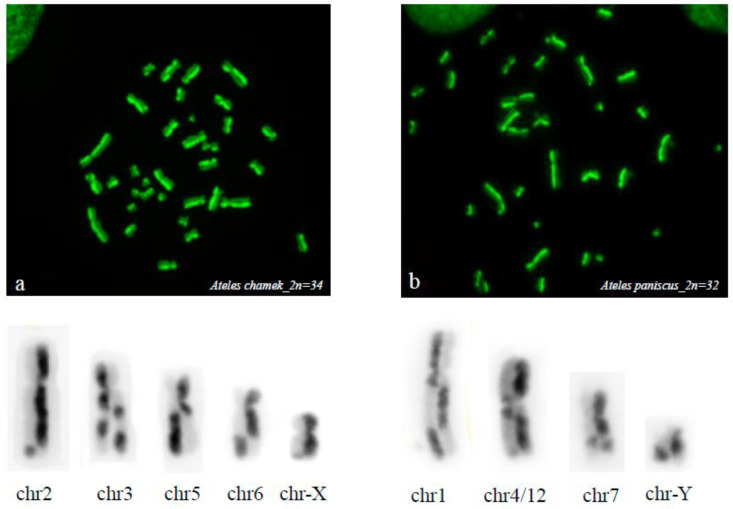
G-SCEs *A. chamek* and *A. paniscus*. Examples of chromosome pairs involved in evolutionary rearrangements for the genus. (**a**) chromosome pairs 1, 2, 3, 4, 5, 6, and 7 in *A. chamek*; and (**b**) chromosome pairs 1, 2, 3, 4/12, 7, and 10 in *A. paniscus*.

As would be expected based on size alone, G-SCEs were most frequently observed in the largest chromosome pairs. Furthermore, within this group, G-SCEs most frequently involved chromosome pairs previously described in evolutionary rearrangements for the genus; e.g., pairs 1, 2, 3, 4, 5, 6, and 7 in *A. chamek* (Figure 3a), and pairs 1, 2, 3, 4/12, 7 y 10 in *A. paniscus* (Figure 3b).

**Figure 4:**
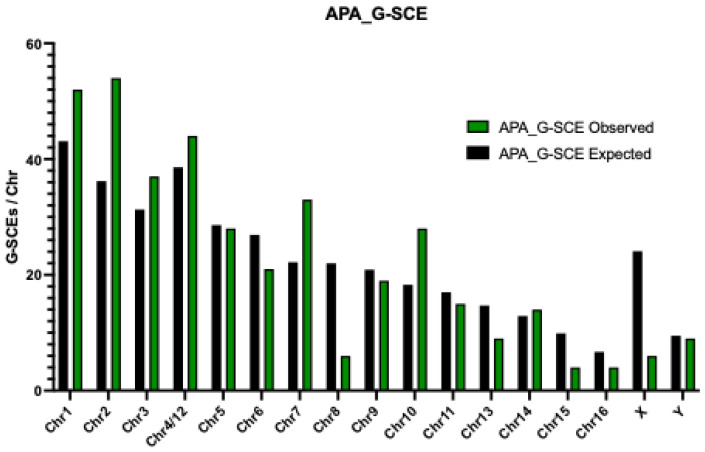
Number of observed (green) and expected (black) G-SCEs for each chromosome pair of *A. paniscus*.

**Figure 5:**
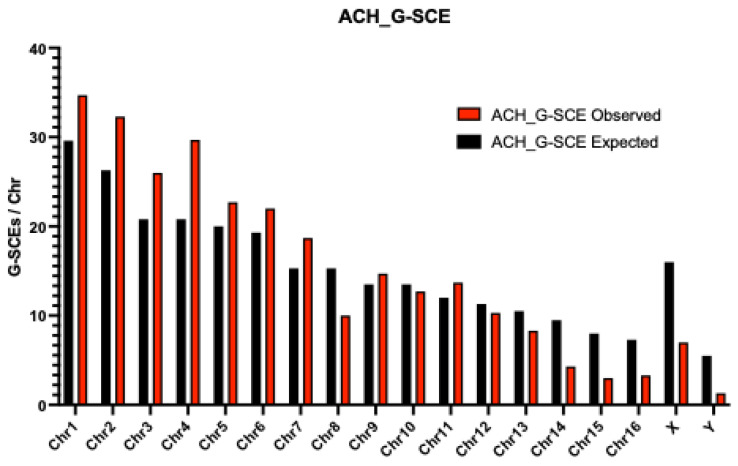
Number of observed (red) and expected (black) G-SCEs for each chromosome pair of *A. chamek*.

Additionally, the Y chromosome of *A. paniscus* displayed a number of G-SCEs, whereas no G-SCEs were observed on the Y chromosome of *A. chamek* (Figure 4 and Figure 5). Statistically significant differences were detected between the observed and expected G-SCE frequencies in both ACH (*p* = 0.046) and APA (*p* < 0.001) (see G-SCE sheets in Supplementary Material).

### 3.3. Analysis of the Telomeric Sister Chromatid Exchange (T-SCE)

**Figure 6:**
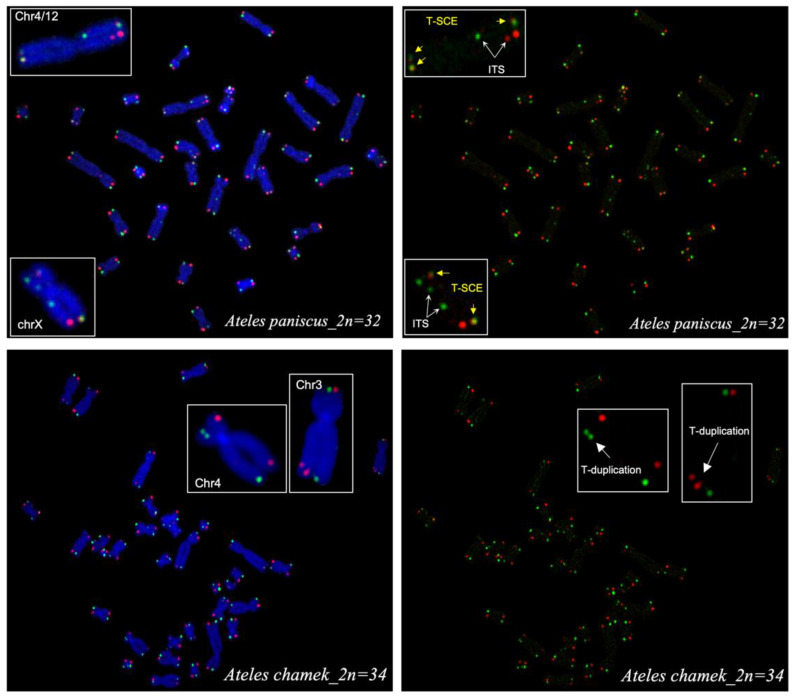
*A. paniscus* and *A. chamek* CO-FISH: Images showing leading (red) and lagging (green) strand telomeres. Metaphase chromosomes counterstained with DAPI (blue) and without. Telomeric-sister chromatid exchanges (T-SCEs), interstitial telomeric sequences (ITSs), and telomere duplications are shown.

CO-FISH detection of telomeres on Ateles metaphases confirmed the expected number of telomeric signals in both *A. paniscus* (64) and *A. chamek* (68) (Figure 6). However, marked differences between the species in the frequencies of telomeric recombination/exchange events (T-SCEs) were observed.

In addition, most metaphases showed more CO-FISH signals than expected for the corresponding ploidy number for leading- and lagging-strand telomeres. A fraction of these additional signals were associated with T-SCEs, while others were involved in telomere duplications and interstitial telomere signals (ITSs). 

**Figure 7:**
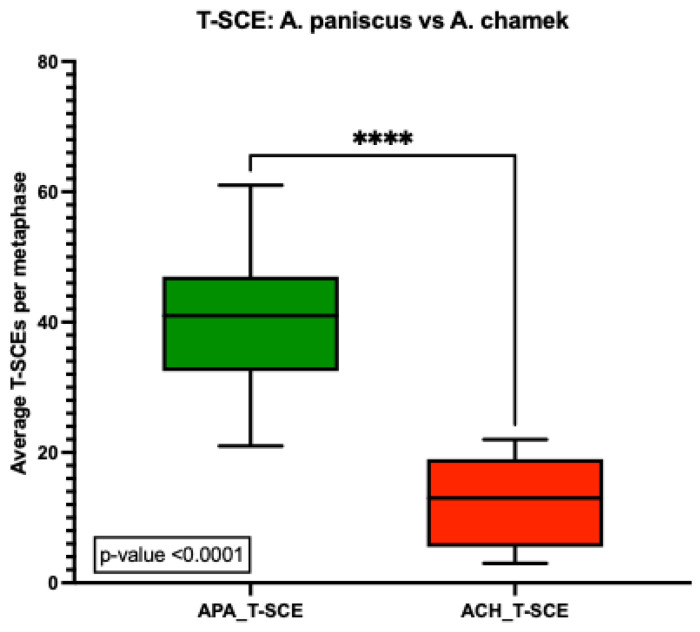
Average number of T-SCEs detected in *A. paniscus* vs. *A. chamek*: The average number of exchanges was significantly higher in *A. paniscus* showing an average of 40 T-SCEs per metaphase compared to 12 T-SCEs per metaphase in *A. chamek* (*p* < 0.0001). Statistical analysis was done using a one-way Anova test (**** *p* < 0.0001).

Interestingly, quantification of T-SCEs showed a high number of telomeric exchanges for both species. The number of T-SCEs observed was higher in *A. paniscus*, with an average of 40 T-SCEs/metaphase compared to 12 T-SCEs/metaphase for *A. chamek*. The difference was statistically significant, with a *p*-value < 0.0001 (Figure 7).

**Figure 8:**
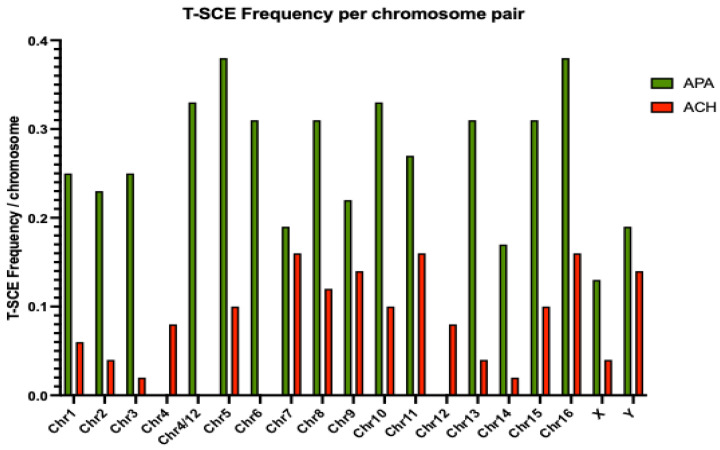
Comparison of the T-SCE frequencies per chromosome observed in *A. paniscus* (green) and *A. chamek* (red). The histogram shows a higher number of telomeric exchanges for all chromosomes of *A. paniscus*, ranging from 0.13 for the X-chromosome, to 0.38 for pairs 5 and 16. For *A. chamek*, T-SCE frequencies ranged from 0.02 for pairs 3 and 14, to 0.16 for pairs 7, 11, and 16.

A more detailed analysis of T- SCE by chromosomal pair revealed differences across the genome between the two species. In *A. chamek*, the highest numbers of T-SCEs observed per chromosome involved chromosome pairs 7, 11, and 16, with a frequency of 0.16, and chromosome pair 9, with a frequency of 0.14. T-SCE frequencies on the X-chromosome were also 0.14. In contrast, the highest frequency of T-SCEs observed in *A. paniscus* was 0.38 for chromosome pairs 5 and 16, and 0.33 for chromosomes 4/12, and 10. Figure 8 shows the complete distribution of the T-SCE frequencies scored in all of the chromosomes for each species (see T-SCE sheets in Supplementary Material).

## 4. Discussion

The genomic response to exogenous or endogenous DNA damage differs depending on the characteristics that define each species. Among the variety of potential factors, heterochromatic regions, genome size, and fragile sites are particularly important [4,29]. Moreover, homologous recombination/exchange events between sister chromatids (SCEs) as a means of repair or “bypassing” spontaneous damage, also contributes to a species ability to maintain genome stability. Telomeric and subtelomeric regions are especially noteworthy in this regard, as they represent a highly repetitive and dynamic region of the genome, and they are essential for preserving chromosome integrity [30,31]. Telomeric regions are also considered critical for the analysis of evolutionary processes [32,33,34]. Therefore, characterizing telomeric function and sequence distribution in the karyotypes of non-human primate species is of great interest in genomic instability and karyotype evolution studies. Determination of spontaneous frequencies of both G-SCEs and T-SCEs provides evidence of a particular genome’s sensitivity to potential stressors impacting the mechanisms of DNA damage response and repair. Given the lack of information on this topic in Neotropical primates, we used SCE frequencies for descriptive and comparative purposes. In the present study, we analyzed the genomic stability of two species of *Ateles* by characterizing the spontaneous induction of G-SCEs and T-SCEs in each. 

### 4.1. G-SCE as a Genomic Instability Biomarker in Non-Human Primates

The replication index (RI) results constitute the first formal determination of cell proliferation kinetics in these non-human primates, significantly contributing to characterization of the species cell cycle. Both species showed RI values close to 2, corresponding to most cultured cells going through a second cycle of cell division (83.6% ACH and 67.01% APA) with a doubling time of ~24 h.

G-SCE analysis showed a high sensitivity and specificity for detecting regions prone to breakage and recombination (in the form of SCE). Thus, G-SCE is a valuable genomic instability biomarker that provides information for comparative evolutionary studies. We observed a G-SCE relative frequency comparatively higher in both species than that previously described for *Alouatta caraya*, *Saimiri boliviensis*, and *Cebus apella paraguayanus* (*Sapajus cay*) [19,20,21,22]. For comparison, it is worth noting that G-SCEs per chromosome in normal human fibroblast is 0.11 [35]. Furthermore, even though the mean G-SCE frequency was lower in *Ateles paniscus* (G-SCEr = 0.15) than in *A. chamek* (G-SCEr = 0.20), the distribution of G-SCEs was highly associated with chromosomal pairs implicated in the evolution of this genus. These results are consistent with the fact that the *Ateles* genome accumulated a high number of evolutionary rearrangements, and with the drastic reduction in the chromosomal number from the ancestor of Atelidae (2n = 62) to the ancestor of *Alouatta, Ateles, Brachyteles* and *Lagothrix* (Atelidae, 2n = 62) to the ancestor of *Ateles* (2n = 34) [17,22]. This chromosomal reduction implied, per se, a critical genomic reorganization that becomes evident in multiple unstable regions. Chromosome pairs 8, 10, 12, 14, 15, and 16 showed G-SCE frequencies considerably lower than expected, given their size (Figure 3 and Figure 4). Statistical analysis indicated that these pairs do not have significantly unstable regions. This was an expected result considering that these pairs, among the smallest pairs of the karyotype, have a high degree of conservation [17]. Notably, chromosome pair number 8 showed G-SCE frequencies markedly lower than expected in *A. chamek* and *A. paniscus*. This result may be associated with the fact that the nucleolar organizer region (NOR) resides in this pair, which could provide some protection against the occurrence of breaks and exchanges [22]. In addition, the lower-than-expected frequency of exchanges observed on the X-chromosome may have an evolutionary cause. Since most of the X-chromosome sequence is ancestral, containing regions retained from the ancestral mammalian autosome that evolved 200–300 million years ago [36]. It has been hypothesized that the presence of structural or epigenetic cues may confer protection on the X-chromosomes against DNA damage and rearrangements [22]. This high degree of conservation is due to its structural stability, as well as its functional importance in terms of sexual determination. The evolutionary relevance of sex chromosomes is even more notable in *Alouatta* spp. due to the presence of a highly complex sexual determination system [37]. Consistently, their multiple sex chromosomes showed the lowest observed SCE frequencies of the *Alouatta caraya* karyotype [22]. Similarly, a low degree of instability was also observed in the X-chromosomes of both species of *Ateles* analyzed in this study. On the contrary, the Y-chromosomes of *A. paniscus* and *A. chamek* both showed the highest SCE frequency among the smaller chromosomes of the complement. Interestingly, the SCE frequency in the Y-chromosome was higher in *A. paniscus* than in *A. chamek*, which is consistent with the interspecies comparative genomic hybridization (iCGH) analysis in *Ateles spp*. reported by Fantini et al. 2016 [26]. In addition, it is noteworthy that previous phylogenetic analysis of *Ateles* rearrangements showed pericentric inversions intra- and inter-species, which may explain the high rate of exchanges [25].

### 4.2. T-SCE as a Telomere Instability Biomarker in Non-Human Primates

Detecting telomere-specific SCE (T-SCE) using the strand-specific methodology of CO-FISH facilitated the evaluation of recombination events within the telomere region. Increased frequencies of T-SCEs have been observed in cancer cells, and in normal cells facing stress conditions [32,35]. Here, we find a high level of spontaneous T-SCEs in both species. However, there is a 3.3-fold difference in the frequencies of T-SCEs in *Ateles paniscus* compared to *A. chamek*. Furthermore, T-SCE quantification showed an average of 40 T-SCEs per metaphase in *A. paniscus*; while, *A. chamek* only showed an average of 12 T-SCEs per metaphase. In addition, upon detailed analysis of T-SCEs per chromosome, we observed frequencies that range from 0.19 to 0.38 in *A. paniscus*. Chromosome pairs 5 and 16 showed the group’s highest frequency of T-SCEs per chromosome, denoting exchanges in 38% of telomeres (three out of eight telomeres per metaphase). In addition, chromosome pairs 4/12, 6, 8, 10, 13, and 15 exhibited a proportion higher than 30% of telomeres presenting T-SCEs. In comparison, *A. chamek* showed an overall lower exchange frequency ranging from 0.02 to 0.16. In this case, chromosome pairs 7, 11, and 16 showed the highest frequency of the group, with 16% of telomeres per chromosome pair showing exchanges. Furthermore, chromosomes 5, 8, 9, 10, and 15 had a proportion higher than 10% of T-SCEs. For comparison, T-SCE frequencies per chromosome were higher (0.27 for *A. paniscus*) and lower (0.09 for *A. chamek*) than the reported normal human dermal fibroblast with 0.24 [35]. Interestingly, the sex chromosomes showed a differential instability in *A. chamek*, where the X-chromosome had a frequency of 0.04, and the Y-chromosome was 0.14. However, in *A. paniscus*, the sex chromosome followed the same higher trend as the autosomal chromosomes, having a frequency of 0.13 and 0.19 for chromosomes X and Y, respectively. Despite this difference, the Y-chromosome always displayed more T-SCEs than the X-chromosome in both species.

### 4.3. Perspectives: The Tale of Two Biomarkers

Considering that formation of SCE is mediated by homologous recombination [38], we proposed an association between the frequency of spontaneous SCE and the presence of evolutionary rearrangements in certain chromosomal regions [22]. That is, regions with higher SCE frequencies would be those experiencing greater instability, and therefore should correspond to chromosomal regions where genome reorganizations occurred during evolution of the different lineages. Previous studies suggest that repetitive sub-telomeric regions of mammalian chromosomes are hot spots for exchanges, as they display high levels of SCE [32]. Furthermore, the proportion of SCE is higher within the telomere repeats themselves, estimated to be ~20 times more than that observed at genomic loci in mouse cells [39] and 1600 times more than in human cells [40]. Interestingly, combining results from G-SCEs and T-SCEs for both monkey species leads to the conclusion that the number of genomic exchanges is inversely proportional to the number of telomeric exchanges. In other words, *Ateles chamek*, showed a high frequency of G-SCEs but a low frequency of T-SCEs; conversely, *Ateles paniscus* showed a low frequency of G-SCEs but a high frequency of T-SCEs. The high frequency of G-SCEs may implicate greater genomic instability over time in *A. chamek*, leading to more genomic reorganization [25].

Moreover, high T-SCE frequencies may have different potential outcomes, one of which could result in more balanced telomere length dynamics over time, thereby facilitating survival, despite the natural telomere shortening that accompanies high T-SCE frequencies [38], as in *A. paniscus*. However, due to spontaneous unequal exchanges, the process may also give rise to chromosomes with both shorter and longer telomeres [39,41]. Therefore, some cells will have chromosomes with long telomeres, overcoming the high levels of T-SCEs. Other cells will have chromosomes with very short telomeres, leading to senescence and, over time, degeneration and cellular aging [42]. 

Taken together, these results suggest that the *Ateles* genome presents a much greater intrinsic instability than other previously analyzed genera, evidenced by the high rate of spontaneous induction of SCEs, particularly at telomeres. We propose that SCE analyses could provide a valuable indicator of instability in *Ateles*, and other species as well. The results presented here are the first description of G-SCEs and T-SCEs in *Ateles.* Furthermore, our analyses are supportive of regions of *Ateles* chromosomes susceptible to recombination and exchange events that are fragile regions and evolutionary hot spots.

## 5. Conclusions

We analyzed genomic instability and compared their respective variability in two species of *Ateles* by characterizing, for the first time, the spontaneous induction of G-SCEs and T-SCEs. Both analyses showed a high sensitivity and specificity for detecting regions prone to breakage and recombination. Thus, we propose SCE analyses as a valuable indicator of genome instability in non-human primates. In addition, the distribution of G-SCEs was highly associated with chromosomal pairs implicated in the evolution of this genus. Taken together, our results support the hypothesis that regions of *Ateles* chromosomes susceptible to recombination events are fragile sites and evolutionary hot spots.

## Figures and Tables

**Table 1:** G-SCE quantification in two species of non-human primates, *A. chamek* and *A. paniscus*. G-SCE/cell: average number of genomic sister chromatid exchange per cell. G-SCEr: number of G-SCEs relative to the total number of chromosomes of all metaphases scored.

Species	G-SCE/Cell	G-SCEr
*Ateles chamek*	6.91	0.203
*Ateles paniscus*	4.78	0.148

## Data Availability

Not applicable.

## Supplementary Materials

The following supporting information can be downloaded at: https://www.mdpi.com/article/10.3390/ani13030510/s1, Nieves et al._supplemental information_rev.xlsx.
